# A Galactosidase-Activatable Fluorescent Probe for Detection of Bacteria Based on BODIPY

**DOI:** 10.3390/molecules26196072

**Published:** 2021-10-08

**Authors:** Xi Chen, Yu-Cong Liu, Jing-Jing Cui, Fang-Ying Wu, Qiang Xiao

**Affiliations:** 1 College of Chemistry, Nanchang University, Nanchang 330031, China; chenxi2016@email.ncu.edu.cn; 2 Key Laboratory of Organic Chemistry in Jiangxi Province, Institute of Organic Chemistry, Jiangxi Science & Technology Normal University, Nanchang 330013, China; liuyucong0522@163.com (Y.-C.L.); jingvsling@126.com (J.-J.C.)

**Keywords:** BODIPY, *β*-galactosidase activity, PET, fluorescent

## Abstract

Pathogenic *E. coli* infection is one of the most widespread foodborne diseases, so the development of sensitive, reliable and easy operating detection tests is a key issue for food safety. Identifying bacteria with a fluorescent medium is more sensitive and faster than using chromogenic media. This study designed and synthesized a *β*-galactosidase-activatable fluorescent probe BOD-Gal for the sensitive detection of *E. coli*. It employed a biocompatible and photostable 4,4-difluoro-3a,4a-diaza-s-indancene (BODIPY) as the fluorophore to form a *β*-*O*-glycosidic bond with galactose, allowing the BOD-Gal to show significant on-off fluorescent signals for in vitro and in vivo bacterial detection. This work shows the potential for the use of a BODIPY based enzyme substrate for pathogen detection.

## 1. Introduction

Foodborne disease is a widespread and ever-increasing public health problem, especially in developing countries and *Escherichia coli (E. coli)*, *Listeria monocytogenes* and *Salmonella* are the most widespread pathogenic microorganisms that contaminate raw foods and drinking water [1]. Individuals infected by pathogenic bacteria show diarrhea, fever and nausea, with neurological disorders, multiple organ failure and death in severe cases [2], so monitoring and control of food pathogens are vitally important.

Although most *E. coli* strains are harmless, some types of pathogenic *E. coli,* such as Shiga toxin-producing *E. coli*, *E. coli* O157 and O104 can be life threatening [3]. As the key to control and prevent foodborne disease, various pathogen detection methods were developed [4] of which one of the most important uses is fluorogenic culture media to identify the pathogens. Fluorogenic media based on specific enzyme substrates have clear advantages over other detection methods [5], as it eliminates the need for subculture and further biochemical testing to establish the identity of certain microorganisms [6], simplifying the identification procedure and shortening the detection time. It is also more sensitive than chromogenic media since the change of light emission is more visually perceptible [7]. The principle of fluorogenic media detection is to design fluorogenic substrates, which can be specifically metabolized by pathogens of interest, resulting in the generation of a new fluorogenic entity through a reversible/irreversible process, causing associated changes in a fluorescence spectrum [8]. As an example, *β*-galactoside and *β*-glucuronide are the two target enzyme substrates which can be metabolized by *E. coli* [9]. The classical molecule 4-methylumbelliferyl-*β*-D-glucuronide (MUG) and its derivatives were designed to detect *E. coli* [8,10,11,12], but it is pH sensitive and its emitting wavelength region is near the autofluorescence of microorganisms [13]. A fluorescein substrate based on the hydrolysis of esterase has also been developed to detect *E. coli*. in drinking water [14], but esterase was metabolized by most of the organisms, thus, its selectivity was a potential problem. There is therefore a need to develop a highly sensitive and specific probe that is non-susceptible to pH change and can be used in fluorogenic media for the pathogens detection.

**Scheme 1:**
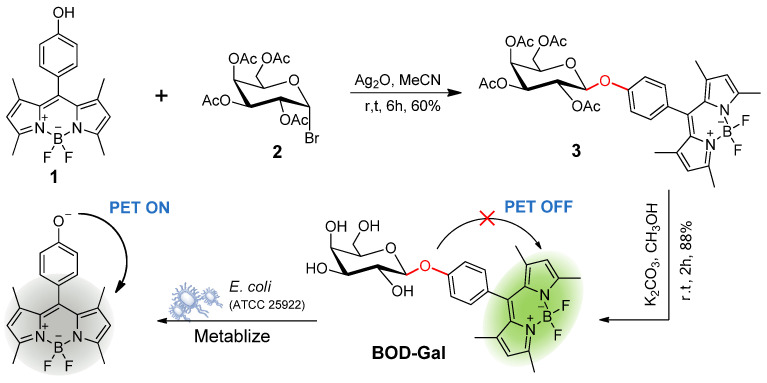
Synthesis Bod-Gal and the proposed sensing mechanism fo*r E. coli* (ATCC 25922).

In this study, a *β*-D-galactosidase (*β*-Gal) activatable fluorogenic probe (BOD-Gal) was developed based on a 4,4-difluoro-3a,4a-diaza-s-indancene (BODIPY) fluorophore (Scheme 1). A BODIPY fluorophore possessing low toxicity and high biocompatibility can be modified in multiple sites and a slightly structural modification can tune its emitting light [15,16]. It has the merits of high extinction coefficient, high quantum yield, excellent photophysical stability and pH resistance [17], so BODIPY fluorophore and *β*-D-galactose can be linked by a *β*-*O*-glycosidic bond, which could be hydrolyzed by *E. coli* producing *β*-Gal and generate BODIPY fluorophore **1** in situ. This phenoxy residue could trigger a strong photoinduced electron transfer (PET) processing in BODIPY dyes [18] and BOD-Gal would show a highly sensitive response to *β*-Gal by an on-off fluorescent response in the PBS buffer. In addition, the PET mechanism was rationalized by the density functional theory (DFT) calculations. When applied to living *E. coli* samples, it can also successfully indicate the presence of pathogens on a media plate.

## 2. Results

### 2.1. In Vitro Spectrum Study

**Figure 1:**
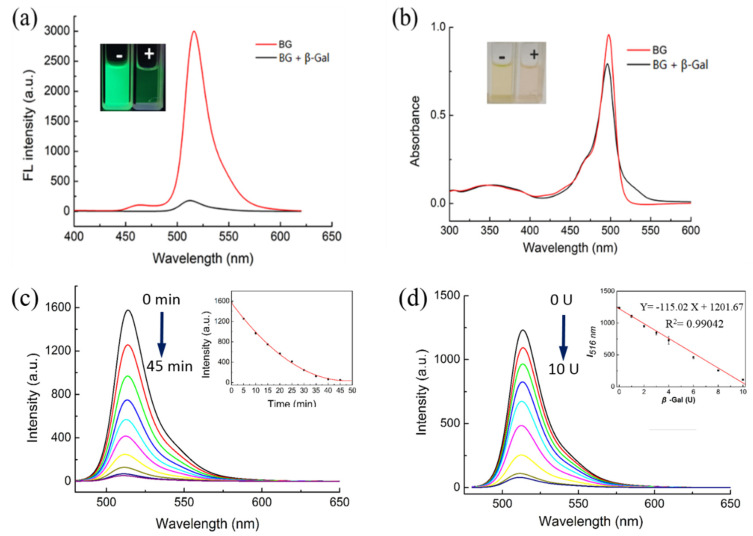
Fluorescence and absorption changes of BOD-Gal to *β-*Gal (8 U) in DMSO/PBS solution (PBS / DMSO = 49:1 *v*:*v*, pH = 7.4). “-” indicated the absence of *β-*Gal, “+” indicated the presence of *β-*Gal. (**a**) Fluorescence changes, λ_ex_ = 470 nm. (**b**) Absorption changes. (**c**) Time dependence of fluorescence spectra (0–45 min, λ_ex_ = 470 nm). Inset: Curve of fluorescence intensity versus time. (**d**) Fluorescence changes of BOD-Gal to different concentration of *β-*Gal (0 U–12 U), λ_ex_ = 470 nm. Inset: The relationship between I_516_ nm and the *β-*Gal concentration.

The sensing ability of BOD-Gal was first evaluated in vitro using *β-*Gal. The fluorescence and absorption responses of BOD-Gal (20 μM) in the absence and presence of *β*-Gal was recorded in aqueous solutions of phosphate-buffered saline (PBS) with dimethyl sulfoxide (DMSO) at a ratio of 49:1 (*v*:*v*) at a pH of 7.4, shown in Figure 1. The BOD-Gal displayed a strong green emission with a maximum at 516 nm, upon excitation at 470 nm. After BOD-Gal was incubated at 37 °C with *β-*Gal for 35 min, the fluorescence was reduced significantly as seen in Figure 1a. The UV spectra of BOD-Gal were investigated in the absence or presence of *β-*Gal. As shown in Figure 1b, the maximum absorption peaks were both at 498 nm, demonstrating that BOD-Gal enabled the assay of *β-*Gal based on a turn-off fluorescence mode.

Considering that a BOD-Gal probe displayed distinct fluorescence response changes to *β-*Gal in aqueous solutions, the influence of incubation time (Figure 1c) and the enzyme concentration (Figure 1d) on BOD-Gal were studied. After 8 U *β-*Gal was added to 10 μM BOD-Gal in PBS solution, the fluorescence intensity decreased with time and became stable after 35 min, indicating that the hydrolysis reaction was complete, so in the subsequent assay, the detection limit was set to 35 min. Fluorescence changes of BOD-Gal to different concentrations of *β-*Gal from 0 to 12 U were also investigated and the emission intensity decreased sharply with the increase of the concentration of *β-*Gal. In addition, fluorescence intensity is linearly correlated to the enzyme concentration in the range of 0 U–10 U (the insert of Figure 1d, R^2^ = 0.9904). The limit of detection (LOD = 3σ/slope) for BOD-Gal toward *β-*Gal was calculated to be 0.038 U/mL.

**Figure 2:**
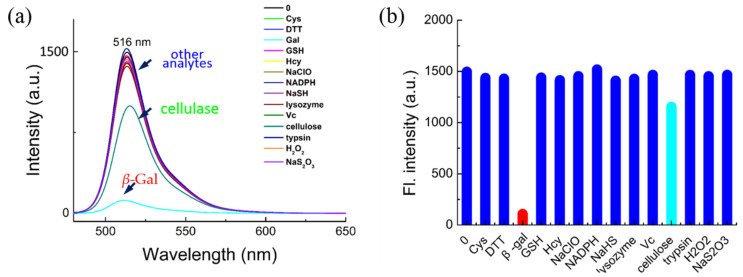
The selectivity of BOD-Gal for *β-*Gal. (**a**) Fluorescence spectra of BOD-Gal to various analytes in an aqueous system (PBS/DMSO = 49:1 *v*:*v*; pH = 7.4, 37 °C) with *β*-Gal (8U), Cys (1 mM), DTT (1 mM), GSH (1 mM), Hcy (1 mM), NaClO (1 mM), NADPH (1 mM), NaHS (1 mM), lysozyme (1 kU), Vc (1 mM), cellulose (1 kU), trypsin (1 kU), H_2_O_2_ (1 mM) and Na_2_S_2_O_3_ (1 mM), λex = 470 nm. (**b**) Corresponding fluorescence intensity.

The potential interference of biological analytes toward BOD-Gal was investigated next. As shown in Figure 2, various enzyme species, amino acids and biomolecules, such as *β-*Gal, cellulase, lysozyme, trypsin, Cys, Hcy, GSH, DTT, NADPH, Vc, NaHS, Na_2_S_2_O_3_, H_2_O_2_ and NaClO, were reacted with BOD-Gal. Only *β-*Gal produced an obvious reduction in fluorescent intensity compared with only subtle changes for up to 100 equiv. of the other competitive analytes, showing that BOD-Gal demonstrated high selectivity for the detection of *β-*Gal.

### 2.2. Kinetics Studies of Enzymatic Reaction

To evaluate the affinity of BOD-Gal toward *β-*Gal, its Km value was calculated by the Hanes–Woolf method. The enzymatic hydrolysis rate was measured by the formation of hydrolysate **1** with high-performance liquid chromatography (HPLC), and the standard curve of compound **1** is shown in Figure S1a. A series concentration of BOD-Gal (5, 10, 15, 20, 25 and 30 μM) was hydrolysed by *β-*Gal in a PBS buffer for 10 min. After inactivation, the samples were submitted for analysis by HPLC, where the reactant BOD-Gal was detected at 2.21 min. A new peak appearing at 10.34 min (Figure S2) was verified as compound **1** through standard sampling (Figure S2). The HPLC results further proved that the sensing mechanism was the hydrolysis of BOD-Gal upon enzyme triggered glycosylic bond cleavage. From the Hanes–Woolf plot (Figure S1b), the Km value was calculated to be 9.5 × 10^−6^ mol/L, which was significantly lower than that of 5-Bromo-4-chloro-3-indolyl-*β*-D-galactoside (X-Gal) at 2.6 × 10^−4^ mol/L [19], showing that BOD-Gal had a much higher affinity for *β-*Gal than commercially available X-Gal.

### 2.3. Theoretical Calculations

**Figure 3:**
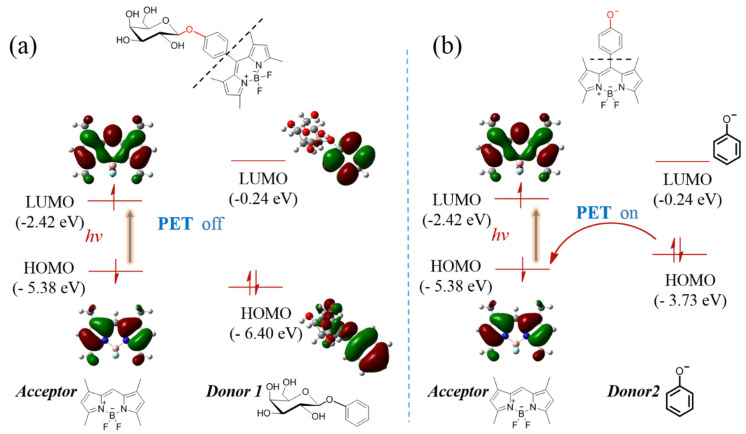
The calculated energy levels of acceptor and donor (**a**) before and (**b**) after hydrolysis reaction according to the theory of the PET mechanism-based on DFT at the B3LYP/6-31 G*(d, p) level.

The DFT theoretical calculations were carried out at B3LYP/6-31 G*(d, p) level using Guassian 16 (considered as gas phase). The molecular structures were divided into “Acceptor” and “Donor” to discuss which was widely used as a computational model for the PET mechanism [20,21]. The results indicated that the highest occupied molecular orbital (HOMO, −5.38 eV) and lowest unoccupied molecular orbital (LUMO, −2.42 eV) energy levels of the acceptor (BODIPY unit) were ranged in between the HOMO (−2.04 eV) and LUMO (−6.40 eV) energy levels of donor **1** (Figure 3a), which implied that intramolecular charge transfer was forbidden, that is, PET progress was off. However, after the glyosidic bond of BOD-Gal was hydrolyzed by *β-*Gal to transform into a phenol anion subunit, the acceptor PET (a-PET) progress could be activated, where the HOMO energy level of donor **2** rose to −3.73 eV between the HOMO and LUMO energy of the acceptor part (Figure 3b) [22]. The optimized geometry and atom list were shown in Figures S3 and S4, Tables S1 and S2. Thus, the theoretical calculations gave a reasonable explanation of fluorescence on-off based on the PET mechanism.

### 2.4. Biological Activity 

Based on the above results, BOD-Gal was applied to a standard formulation of growth media, where petri dishes were poured and inoculated with *E. coli.*

#### 2.4.1. Biological Toxicity to Bacterial Propagation

**Figure 4:**
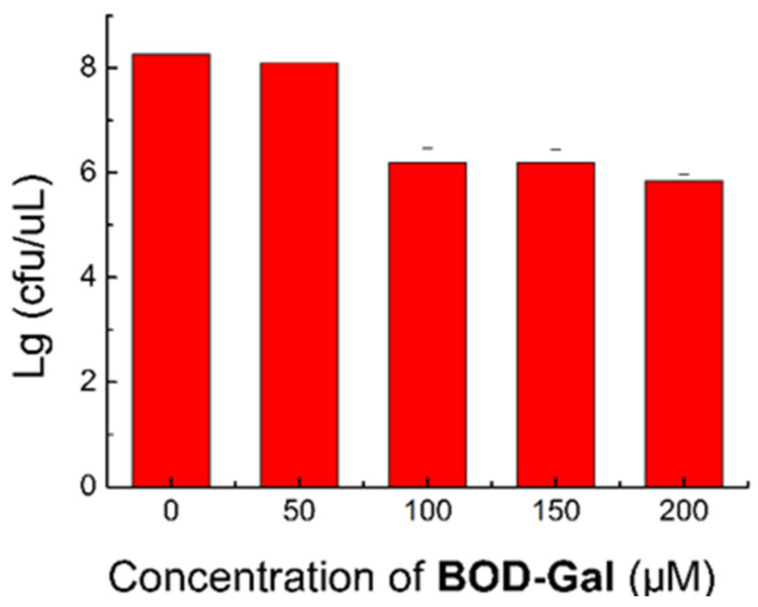
Standard plate count method (SPC) assay of *E. coli* was treated in the presence of BOD-Gal (0–200 μM) incubated for 8 h.

To assess its biological toxicity and biocompatibility, the influence of various concentrations of BOD-Gal (0, 50, 100, 150 and 200 μM) on the growth of bacteria was investigated. The colony numbers are shown in Figure 4 and the corresponding data are shown in Table S3. When cultured at 50 μM, the growth of *E. coli* was not affected, but at higher concentrations (100, 150 and 200 μM), the growth rates were reduced to about 70%. This data indicated that the concentration of BOD-Gal had better not exceed 50 μM in bacterial culturing.

#### 2.4.2. Fluorescence on Agar

**Figure 5:**
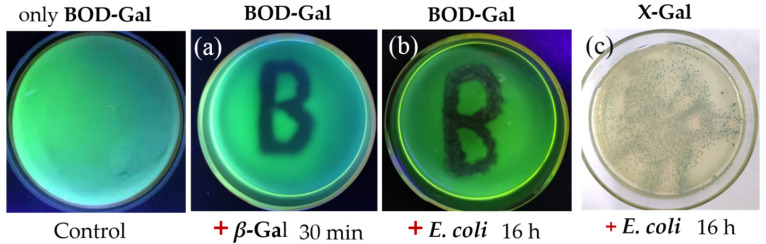
Comparison of different substrates on plate. (**a**) Painting *β*-Gal and (**b**) Inoculating *E. coli* (ATCC 25922) on the plate containing BOD-Gal. (**c**) Inoculating *E. coli* on the plate containing X-Gal (267 μg/mL). UV irradiation was achieved using a hand-held UV lamp, X-Gal staining appearance was in the ambient light condition.

The sensing effects of the enzyme (*β*-Gal) and pathogenic *E. coli* were compared on LB agar containing BOD-Gal. X-Gal was selected as the control, which is a widely used substrate for blue/white selection of *β*-Gal in laboratory and bioengineering. The results are shown in Figure 5.

The *β*-Gal was initially painted on the LB agar containing BOD-Gal. After culture at 37 °C for 30 min, the green fluorescence of the *β*-Gal-exposed region had significantly faded under UV illumination at 365 nm (Figure 5a), which indicated that BOD-Gal could be rapidly hydrolyzed by *β*-Gal on the agar medium. Next, *E. coli* (ATCC 25922) was inoculated on the agar that contained the BOD-Gal and X-Gal, respectively, and incubated at 37 °C for 16 h. From Figure 5b,c, it showed that the plate with BOD-Gal was more clearly distinguished under visual inspection, showing that BOD-Gal exhibited more sensitive detection than traditionally used X-Gal.

## 3. Materials and Methods

### 3.1. General Information

Unless otherwise stated, all the reagents were obtained from commercial sources and used as received without further purification. The stock solution of BOD-Gal was prepared in DMSO at the concentration of 1 mM. *β*-Gal and the other analytes were dissolved in deionized water and diluted to required concentrations. The NMR spectra were acquired on an AM-400 spectrometer (Bruker Co., Ltd., Karlsruhe, Germany) at room temperature with CDCl_3_ or DMSO-d_6_ as the solvent and TMS used as an internal standard. High-resolution mass spectrometry data were performed with an AB SCIEX TOF 4600 (AB Sciex Pte. Ltd., Framingham, MA, USA). Fluorescence spectra measurements were recorded on an F-7100 fluorescence spectrophotometer (Hitachi, Ltd., Tokyo, Japan) and UV/Vis spectra measurements were recorded on a UV-2501 spectrometer (Shimadzu Co., Ltd., Yamanashi, Japan). High-performance liquid chromatography (HPLC) analysis was performed on an Agilent 1220 Infinity (Agilent Technologies Inc., Santa Clara, CA, USA).

### 3.2. Enzyme Assay In Vitro

The BOD-Gal was used at a final concentration of 20 μM unless noted. Absorption and fluorescence spectra of BOD-Gal with *β*-Gal sourced from *E. coli* (Sangon Biotech Co., Ltd., Shanghai, China) were performed at 37 °C in a 2 mL total volume of a PBS buffer of 0.2 M at pH of 7.4 with a 1 cm cuvette. 

### 3.3. HPLC Analysis

A series of different concentrations (5, 10, 15, 20, 25, 30, 35, 40 μM) of BOD-Gal was hydrolyzed by 0.8 U *β*-Gal in a PBS buffer at 0.2 M and pH = 7.4 for 10 min at 37 °C, then inactivated in a 100 °C water bath for 1 min. The samples were prepared by adding equal volumes of DMSO into the reaction solution, to completely dissolve the components. The samples were analysed by HPLC at ambient temperature, using water and acetonitrile as the mobile phase at a ratio of 48:52 (*v*:*v*) and detected at 496 nm. The peak corresponding to BOD-Gal and hydrolysate **1** was integrated (Figure S1).

### 3.4. Biological Experiment

#### 3.4.1. The Culture of Bacteria 

The *E. coli* (ATCC 25922) were inoculated from frozen stock into the sterile LB broth, by adding 2.5 g LB broth powder into 100 mL deionized water, autoclaving at 120 °C for 20 min and left at room temperature (r.t.) in a shaken flask. Depending on the experiments, after 8 to 16 h of vigorous shaking at 150 rpm at 37 °C in the dark, 200 μL isopropyl-beta-D-thiogalactopyranoside (IPTG) at a concentration of 0.1 g/L in sterilized deionized water was added and remixed by shaking at 37 °C to induce E. coli expressing *β*-Gal.

#### 3.4.2. Preparation of Culture Media

The plate was prepared by adding 2 g agar (Solarbio and Technology Co., Ltd., Beijing, China) and 2.5 g LB broth (Hope Bio-Technology Co., Ltd., Qingdao, China) into 100 mL deionized water, autoclaving at 120 °C for 20 min and leaving to cool at 50 °C. Depending on the experiment, various volumes of stock solution of BOD-Gal that had been filtered through 0.22 μM Millipore were added to the warm agar to obtain the different concentrations of BOD-Gal in agar and then poured into sterile petri plates and cooled at room temperature over night to coagulate. 

#### 3.4.3. Antibacterial Evaluation

The antibacterial activity of BOD-Gal was studied by the standard plate count method (SPC). The original suspension bacterial solution was initially diluted to 10^−2^ dilution and plated on the culture media at different concentrations of BOD-Gal (0, 50, 100, 150 and 200 μM), respectively, with each concentration performed three times in parallel. This process was repeated for the 10^−4^ and 10^−6^ dilutions. The plates were inverted, incubated at 37 °C for 8 h and the petri plates containing between 30 to 300 colonies counted via a colony counter (Icount 20, Shineso, Hangzhou, China). 

### 3.5. Synthesis

The synthesis of BOD-Gal is shown in Scheme 1. The starting material, compound **1**, was synthesized by a routine procedure used in the construction of the BODIPY core [23]. The tetra-*O*-acetyl-galactose bromide **2** was obtained according to the previous method [24]. Compound **3** was synthesized with a 60% yield by Koenigs–Knorr glycosylation of compound **1** and **2**. Zemplén deprotection in all the acetates with K_2_CO_3_/CH_3_OH gave BOD-Gal in 88% yield. All intermediates and BDBH were well characterized by ^1^H NMR spectroscopy, ^13^C NMR spectroscopy and high-resolution electrospray ionization mass spectrometry (HR-ESI-MS).

#### 3.5.1. Synthesis of Compound **1**

4-Hydroxybenzaldehyde (0.49 g, 4 mmol) and 2,4-dimethylpyrrole (0.76 g, 8 mmol) were dissolved in anhydrous CH_2_Cl_2_ (600 mL). Two drops of trifluoroacetic acid (TFA) were added and the resulting mixture was stirred in the dark for 12 h under N_2_ at room temperature. After TLC showed the complete consumption of aldehyde, 2, 3-dichloro-5, 6-dicyano-1, 4-benzoquinone (DDQ) (1.09 g, 4.8 mmol) was added. After the mixture was stirred for 1 h, diisopropylethylamine (DIPEA, 5 mL) and BF_3_·OEt_2_ (5 mL) were added. The resulting mixture was further stirred for another 1 h, then concentrated and filtered. After the filtrate was washed twice with water and brine, the organic layer was collected, dried over anhydrous MgSO_4_ and concentrated under reduced pressure. The obtained crude product was purified by column chromatography (R_f_ = 0.2, PE/EA = 3:1, eluent: PE/EA = 30/1–4/1, *v*/*v*) to give compound **1** (0.38 g, 28% yield) as a yellow-red powder. ^1^H NMR (400 MHz, CDCl_3_) δ (ppm): 7.12 (d, *J* = 8.4 Hz, 2H), 6.94 (d, *J* = 8.4 Hz, 2H), 5.98 (s, 2H), 5.30–5.26 (m, 1H), 2.55 (s, 6H), 1.44 (S, 6H); ^13^C NMR (100 MHz, CDCl_3_) δ (ppm): 156.3, 155.3, 143.2, 141.8, 131.8, 129.4, 127.2, 121.2, 116.1, 14.6; ^19^F NMR (376 MHz, CDCl_3_) δ (ppm): −146.06 (m, 2F). HRMS-ESI (*m*/*z*): [M]^ˉ^ Calc. for (C_19_H_18_BF_2_N_2_O), 339.1480, found: 339.1489.

#### 3.5.2. Synthesis of Compound **3**

Compound **1** (98 mg, 0.3 mmol), tetra-*O*-acetyl-α-D-galactose bromide **2** (148 mg, 0.36 mmol), and Ag_2_O (104 mg, 0.45 mmol) were suspended in dry acetonitrile (5 mL). After the mixture was stirred for 6 h at r.t under argon and filtered, the solvent was removed in vacuum. The residue was purified by silica gel column (R_f_ = 0.5, PE/EA = 2:1, eluent: PE/EA =10/1–3/1, *v*/*v*) to give compound **3** (121 mg, 60% yield) as an orange solid. ^1^H NMR (400 MHz, CDCl_3_) δ (ppm): δ 7.19 (d, *J* = 8.4 Hz, 2H), 7.12 (d, *J* = 8.4 Hz, 2H), 5.97 (s, 2H), 5.54 to 5.52 (m, 1H), 5.48 (d, *J* = 3.2 Hz,1H), 5.16 to 5.12 (m, 2H), 4.28 to 4.23 (m, 1H), 4.18 to 4.09 (m, 2H), 2.53 (s, 6H), 2.18 (s, 3H), 2.10 (s, 3H), 2.04 (s, 3H), 2.02 (s, 3H), 1.39 (s, 6H); ^13^C NMR (100 MHz, CDCl_3_) δ (ppm): 170.36, 170.26, 170.17, 169.40, 157.46, 155.68, 143.08, 141.06, 131.70, 129.94, 129.52, 121.38, 117.61, 99.59, 71.36, 70.85, 68.73, 66.96, 61.45, 20.83, 20.71, 20.65, 14.68; ^19^F NMR (376 MHz, CDCl_3_) δ (ppm): −146.17 (m,2F). HRMS-ESI (*m*/*z*): [M+H]^+^ Calc. for (C_33_H_38_BF_2_N_2_O_10_), 671.2588, found: 671.2587.

#### 3.5.3. Synthesis of BOD-Gal

Compound **3** (78 mg, 0.1 mmol) was dissolved in anhydrous methanol (3 mL), then K_2_CO_3_ (2 mg, 0.02 mmol) was added. The resulting mixture was stirred at r.t for 2 h and the pH was adjusted to 6~7with 1 M aqueous HCl. After the reaction mixture was filtered, the filtrate was concentrated in vacuum. The residue was purified by silica gel column (R_f_ = 0.3, DCM/MeOH = 5:1, eluent: DCM/MeOH = 15:1, *v*/*v*) to afford BOD-Gal (52 mg, 88% yield, m.p. 143–145 °C) as an orange powder. ${\left[\alpha \right]}_{D}^{25}$ = +33.33. ^1^H NMR (400 MHz, DMSO-*d_6_*) δ (ppm): δ 7.26 (d, *J* = 8.4 Hz, 2H), 7.20 (d, *J* = 8.4 Hz, 2H), 6.17 (s, 2H), 5.23 (s, 1H), 4.90 (d, *J* = 8.0 Hz, 2H), 4.67 (s, 1H), 4.54 (s, 1H), 3.72 (s, 1H), 3.63–3.43 (m, 5H), 2.44 (s, 6H), 1.40 (s, 6H); ^13^C NMR (100 MHz, DMSO-*d_6_*) δ (ppm): 156.25, 154.66, 142.70, 142.01, 131.03, 128.98, 127.06, 121.26, 116.84, 101.11, 75.52, 73.23, 70.35, 68.06, 60.29, 14.19; ^19^F NMR (376 MHz, DMSO-*d_6_*) δ (ppm): −143.57 (m,2F). HRMS-ESI (*m*/*z*): [M+Na]^+^ Calc. for (C_25_H_30_BF_2_N_2_O_6_Na), 525.1984, found:525.1959.

## 4. Conclusions

This study designed, synthesized and characterized a novel fluorescent substrate, BOD-Gal, for the detection of *β*-galactosidase activity. It showed a distinct fluorescence reduction after hydrolysis by *β*-Gal, when other biologically competitive enzyme species, amino acids, and small molecules caused only faint fluorescent change. The results of HPLC chromatography confirmed that BOD-Gal was exclusively dissociated by *β*-Gal to a phenoxy residue, which induced a PET mechanism. In addition, BOD-Gal displayed a higher affinity and faster response to *β*-Gal than commercial X-Gal as shown on a Hanes–Woolf plot. For bacteria detection, BOD-Gal also had a significant response to pathogenic *E. coli* on an agar growth medium with low toxicity. In view of its convenience, sensitivity and speed, this substrate has the potential to promote the development of fluorescent media in pathogen detection.

## Data Availability

Not applicable.

## Supplementary Materials

The following are available online. Scheme S1, Figure S1–S11, Table S1–S3.

## Sample Availability

Samples of all the compounds are available from the authors.
