# Supplementary material for: A Galactosidase-Activatable Fluorescent Probe for Detection of Bacteria Based on BODIPY
Source: Molecules. 2021 Oct 8;26(19):6072. doi: 10.3390/molecules26196072 (PMC8512000; doi:10.3390/molecules26196072)
Supplement: Supplementary file 1 [file molecules-26-06072-s001.zip › molecules- ESI 1001.pdf]

molecules

Supporting information

A Galactosidase-Activatable Fluorescent Probe for Detection of Bacteria Based on BODIPY

Xi Chen [1,2], Yu-Cong Liu [2], Jing-Jing Cui [2], Fang-Ying Wu [1,*] and Qiang Xiao [2,*]

[1] College of Chemistry, Nanchang University, Nanchang 330031, China; chenxi2016@email.ncu.edu.cn
[2] Key Laboratory of Organic Chemistry in Jiangxi Province, Institute of Organic Chemistry, Jiangxi Science & Technology Normal University, Nanchang 330013, China; liuyucong0522@163.com (Y.-C.L.); jingvsling@126.com (J.-J.C.)
* Correspondence: fywu@ncu.edu.cn (F.-Y.W.); xiaoqiang@tsinghua.org.cn (Q.X.);
Tel./Fax: +0086-791-83969882 (F.-Y.W.); +0086-791-86422903 (Q.X.)

Supporting Information

Contents

1. **Synthesis of Compound 2……….………………………………………………………........S2**

2. **Standard Curve of 1 and the Hanes-Woolf Plot of Hydrolysis Reaction of BOD-Gal with β-Gal Using HPLC Chromaography…………………………….S3**

3. **HPLC Chromatography of Hydrolysis Reaction Mixture ……………………..…...S3**

4. **Theoratical Calculation Details…………………………………...…………...S4**

5. **Colony Number in SPC Assay……………………………………………….……..S7**

6. **Characterization of Intermediate Compounds and BOD-Gal…………..…..S8-S12**

1. Synthesis of Compound 2

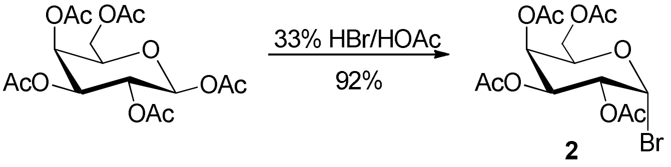

Scheme S1. Synthesis compound **2**.

1,2,3,4,6-tetra-*O*-acetlyl-*β*-D-galactose (50g, 128 mmol) and 33% HBr in acetic acid (125 mL) were added to a 500 mL round botton flask. The mixture was stirred under ice bath for 3h until all solid was completely dissolved. The reaction bottle was left in refrigerate overnight. Then, the reaction mixture was poured into ice water (500mL), extracted with dichloromethane (500mL) and washed with saturated $NaHCO_3$ until neutral. After dried by anhydrous $MgSO_4$ and filtered, the solvent was removed by evaporation to obtain the desired product as a white solid (48.31 g, 92% yield).

2. Standard Curve of 1 and the Hanes-Woolf Plot of Hydrolysis Reaction of BOD-Gal with β-Gal Using HPLC Chromaography

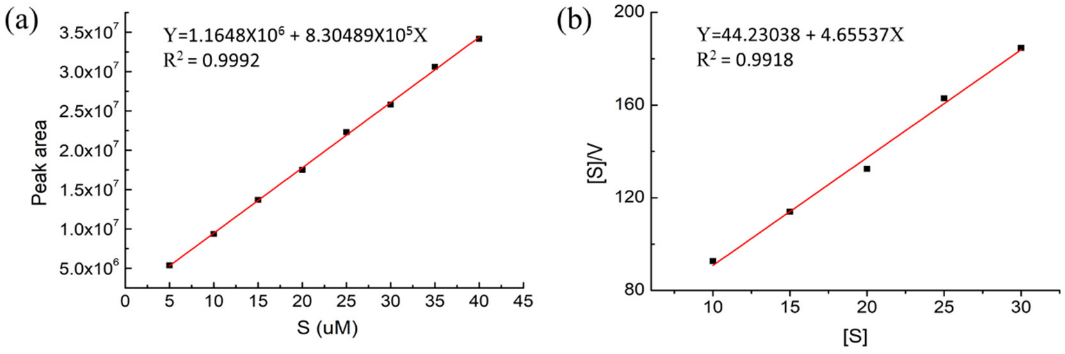

Figure S1. (**a**) Standard curve for compound **1**. Y-axis was peak area, X-axis was concentration of compound **1**. (**b**) Hanes-Woolf plot of hydrolysis reaction of **BOD-Gal** with β-Gal.

3. HPLC Chromatography of Hydrolysis Reaction Mixture

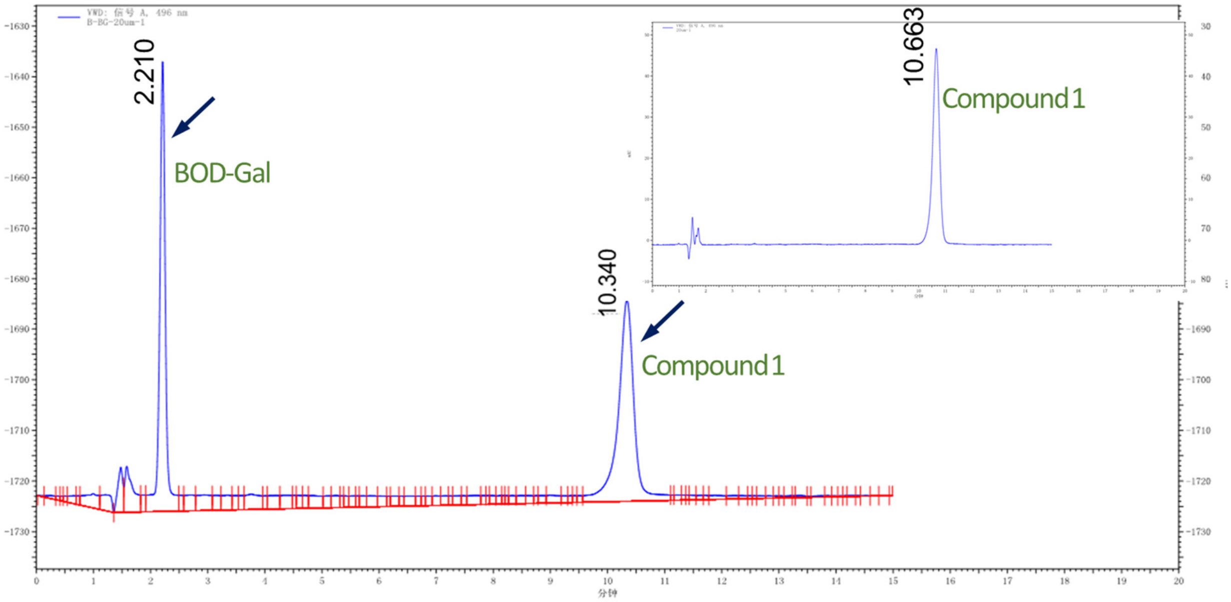

Figure S2. HPLC chromaography of hydrolysis reaction mixture (20 μM BOD-Gal hydrolyzed by β-Gal for 10min). Eluent solvent: H_2O/MeCN (v:v, 48:52), flow rate = 1 mL/min, detection wavelength at 496 nm. Insert is the HPLC chromaography of pure compound **1**.

4. Theoratical Calculation Details

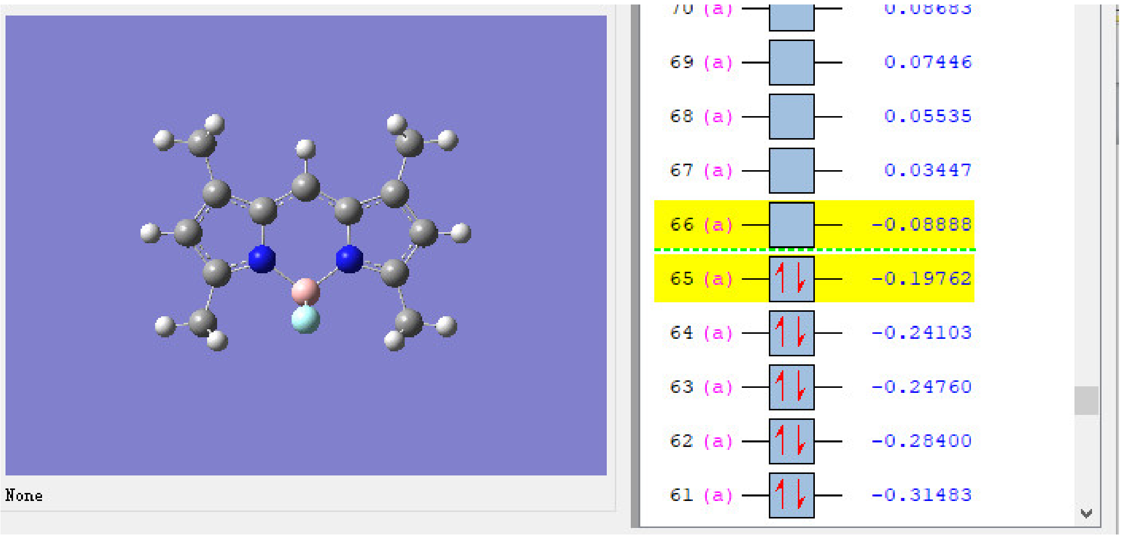

Figure S3. Orbital energy levels of optimized BODIPY unit (Acceptor part).

Table S1. Atom list of optimized BODIPY unit (Acceptor part).

0 1			
C	−0. 00005100	1. 66743100	−0. 00008900
C	−1. 21625400	0. 99441800	0. 00004200
N	−1. 25324700	−0. 40146400	0. 00024800
B	0. 00007700	−1. 34146300	−0. 00053400
N	1. 25322800	−0. 40143400	0. 00040600
C	1. 21621000	0. 99445500	0. 00003700
C	−2. 55557100	1. 48441800	0. 00017000
C	−3. 37286900	0. 36041200	0. 00040500
C	−2. 54439000	−0. 78709800	0. 00041100
C	2. 54442900	−0. 78703200	0. 00056700
C	3. 37283600	0. 36049000	0. 00040200
C	2. 55546800	1. 48449000	0. 00004200
F	0. 00007900	−2. 13154400	−1. 14593200
F	0. 00000600	−2. 13362100	1. 14347200
C	2. 97529400	2. 92285000	−0. 00043800
C	2. 94790700	−2. 22417900	0. 00087200
C	−2. 97539300	2. 92277500	0. 00029200
C	−2. 94779500	−2. 22426800	0. 00017100
H	−0. 00006400	2. 75296200	−0. 00029500
H	−4. 45464600	0. 34480800	0. 00061100
H	4. 45461500	0. 34496000	0. 00058800

H	2.60339200	3.45421900	-0.88428900
H	2.59891100	3.45617100	0.88031300
H	4.06475000	3.00751900	0.00220400
H	4.03528300	-2.32052200	0.00089300
H	2.53886700	-2.73432000	0.87857600
H	2.53891700	-2.73461800	-0.87669400
H	-4.06485200	3.00745200	-0.00124500
H	-2.60253900	3.45464600	0.88343500
H	-2.59994500	3.45558300	-0.88117200
H	-2.53610000	-2.73541500	0.87602000
H	-4.03516200	-2.32068900	0.00319200
H	-2.54138500	-2.73364900	-0.87923100

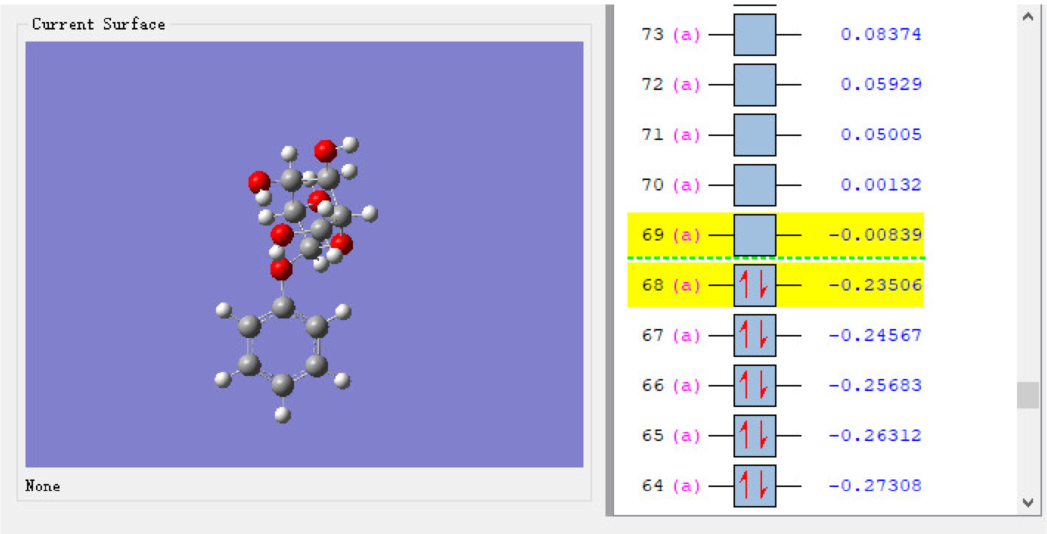

Figure S4. Orbital energy levels of optimized galactose unit (Donor part 1).

Table S2. Atom list of optimized galactose unit (Donor part 1).

	0 1		
O	-0.73177700	0.29837600	0.55444400
C	-2.07667100	0.14826200	0.23724900
C	-2.95291700	0.08313600	1.32372200
C	-4.31652400	-0.08759900	1.09679100
C	-4.80940200	-0.18524700	-0.20694200
C	-3.92383600	-0.12196300	-1.28209500
C	-2.55145200	0.03612500	-1.07238300
O	0.54841100	0.04988400	-1.41035600
C	0.12090000	0.93691300	-0.41092000
C	2.82648500	-0.09329700	-0.50883900
C	1.63454200	-0.85933500	-1.12982800
O	2.24628300	0.03555600	1.91400900
O	1.66117800	2.68953400	-0.54647200
C	1.18211200	-2.14239500	-0.42288500
C	1.25309400	1.65196200	0.34737000
C	2.45415100	0.74557300	0.72302600
O	0.75248200	-1.99806600	0.92460000
O	3.88753600	-0.97170100	-0.16491000
H	-2.55213400	0.17584300	2.32775700
H	-4.99616400	-0.13581500	1.94240900
H	-5.87303800	-0.31341300	-0.38134500
H	-4.29568300	-0.20745700	-2.29897300
H	-1.86185900	0.04583800	-1.90786900
H	-0.46572100	1.69414700	-0.93739600
H	3.13934700	0.63591200	-1.26949200
H	1.94740300	-1.18730400	-2.13100800
H	1.81430600	-0.81877900	1.70888300
H	2.33194800	3.21664600	-0.09280900
H	0.38821400	-2.59744800	-1.03113700
H	2.03281100	-2.82838900	-0.40014000
H	0.82925500	2.05782000	1.27471600
H	3.30511100	1.41596900	0.91612500
H	0.00232600	-1.37468100	0.92739800
H	4.29806800	-1.28035800	-0.98324200

5. Colony Number in SPC Assay

Table S3. Colony number in SPC assay corresponding to Figure 2.

	Counting Numbers				
	0uM	50 uM	100 uM	150 uM	200 uM
**1 th**	7.2×10^{7}	3.2×10^{8}	7.6×10^{5}	2.4×10^{6}	9.2×10^{5}
**2 th**	2.5×10^{8}	1.3×10^{8}	2.4×10^{6}	2.0×10^{6}	7.2×10^{5}
**3 th**	3.6×10^{8}	5.1×10^{7}	2.2×10^{6}	8.4×10^{5}	5.2×10^{5}
**average**	2.3×10^{8}	1.7×10^{8}	1.8×10^{6}	4.3×10^{6}	7.2×10^{5}

6. Characterization of Intermediate Compounds and BOD-Gal

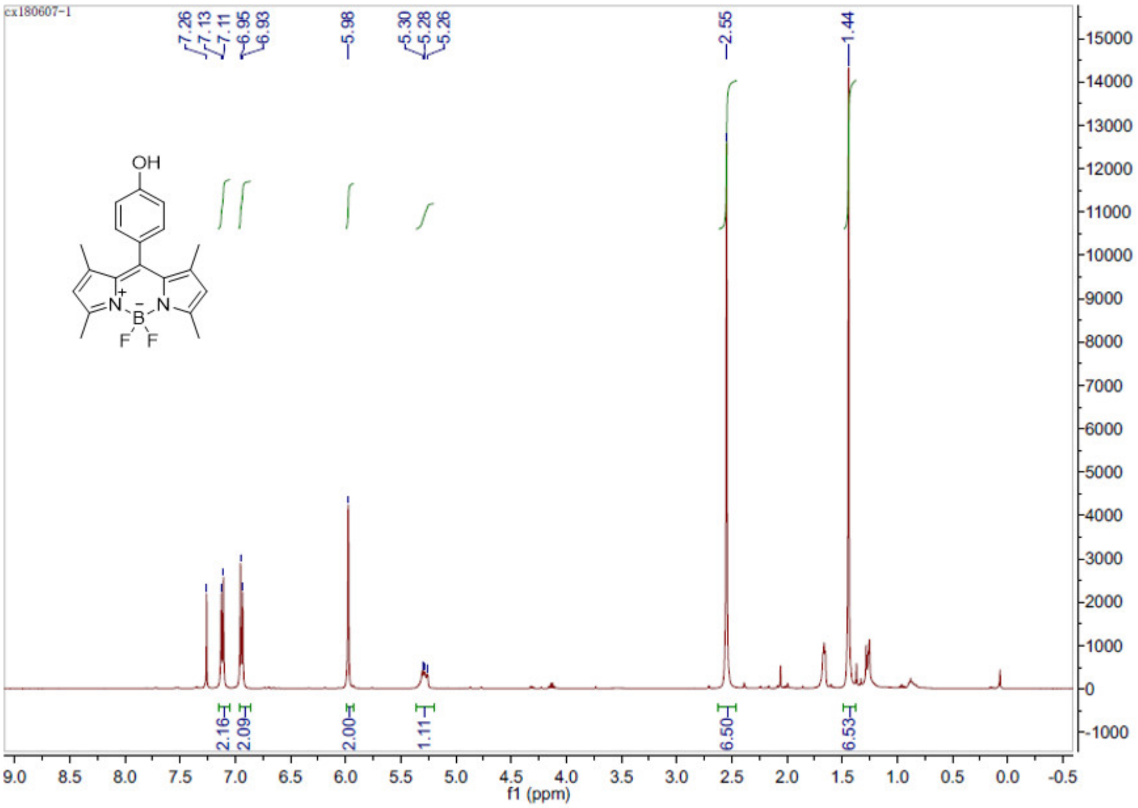

Figure S5. ^{1}H NMR spectrum of compound **1** in $CDCl_3$.

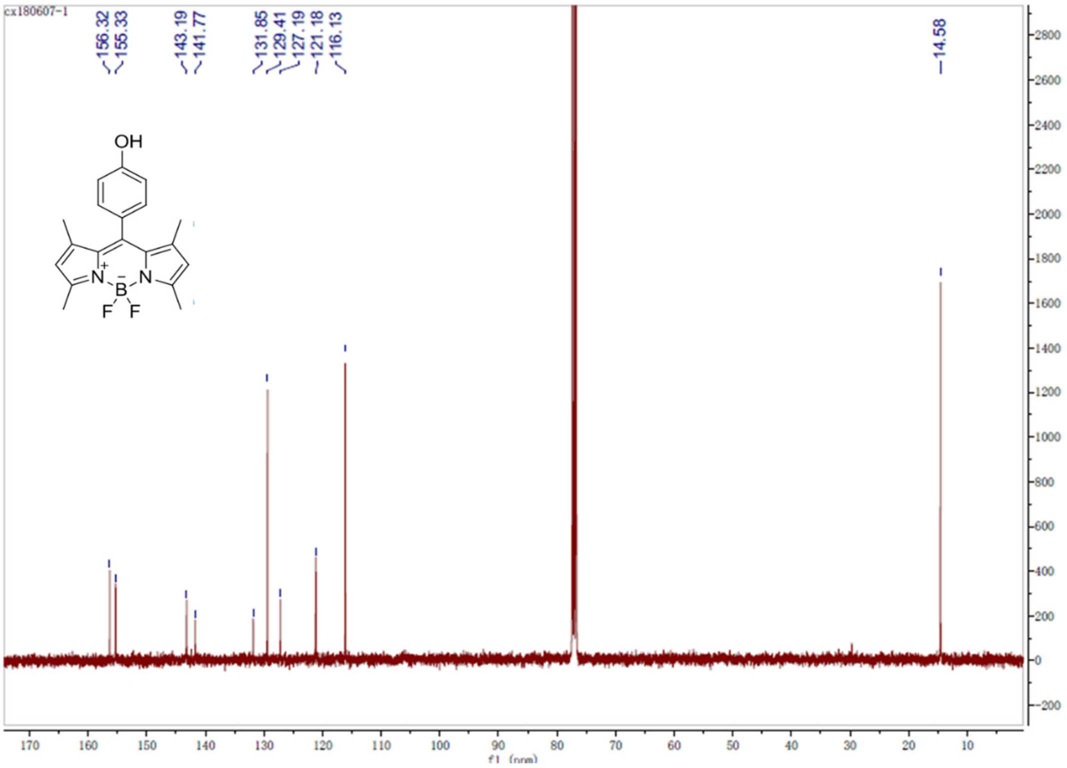

Figure S6. ^{13}C NMR spectrum of compound **1** in $CDCl_3$.

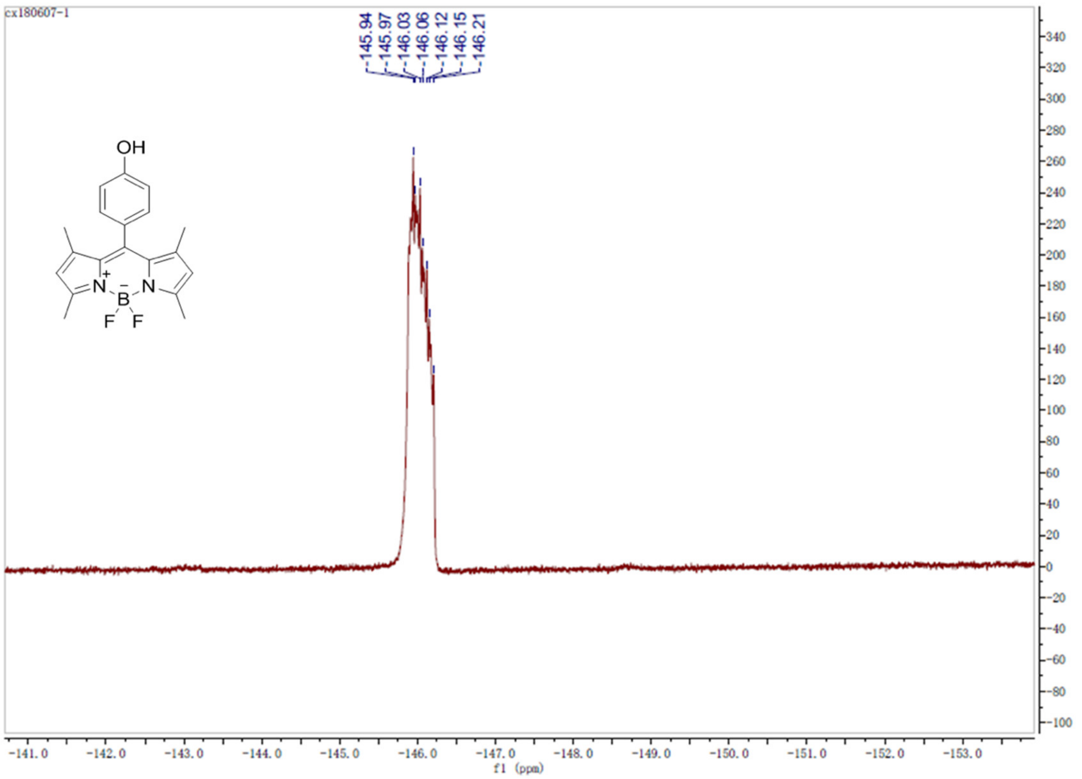

Figure S7 ^{19}F NMR spectrum of compound **1** in $CDCl_3$

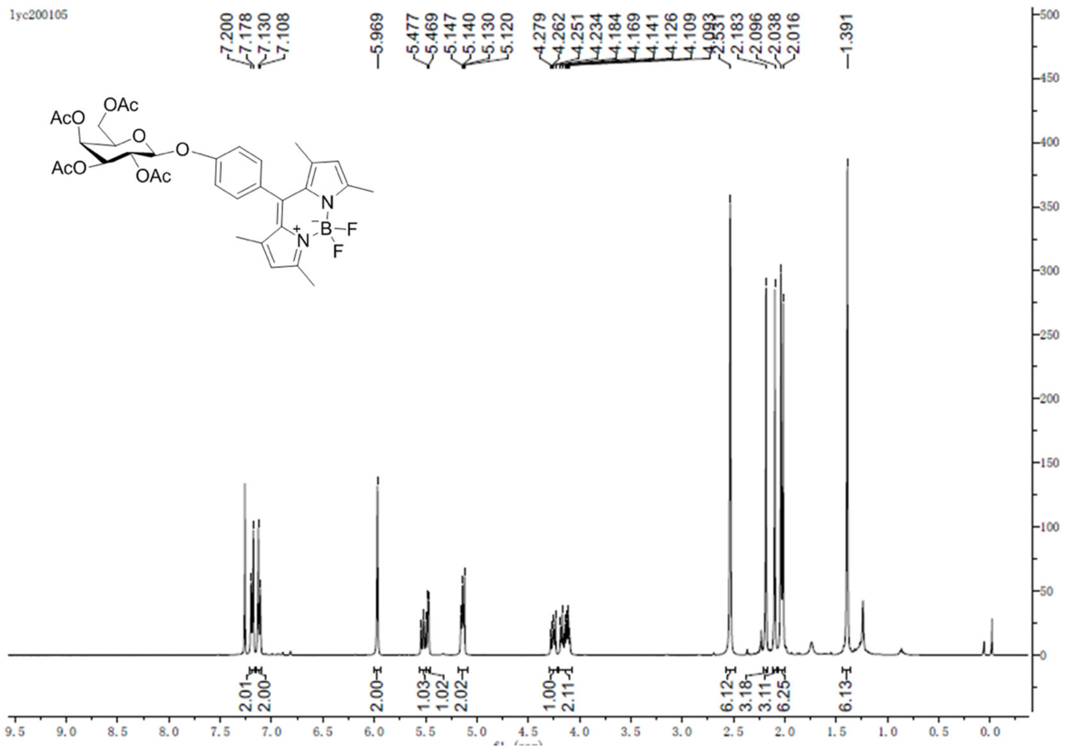

Figure S8 ^{1}H NMR spectrum of compound **3** in $CDCl_3$

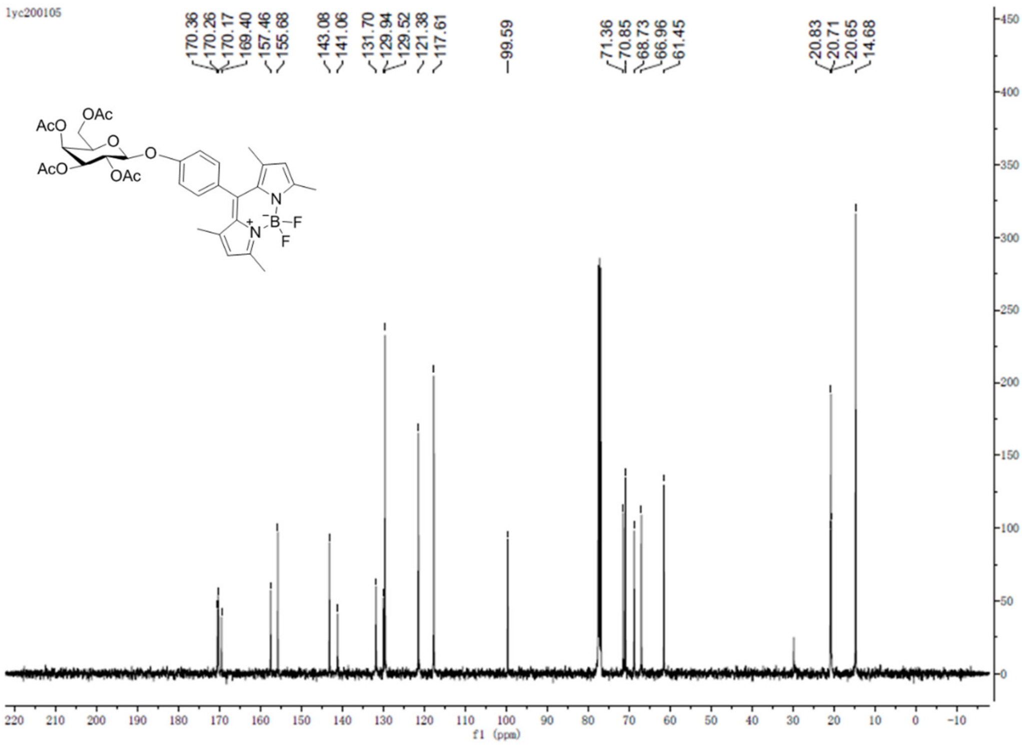

Figure S9 ^{13}C NMR spectrum of compound **3** in $CDCl_3$

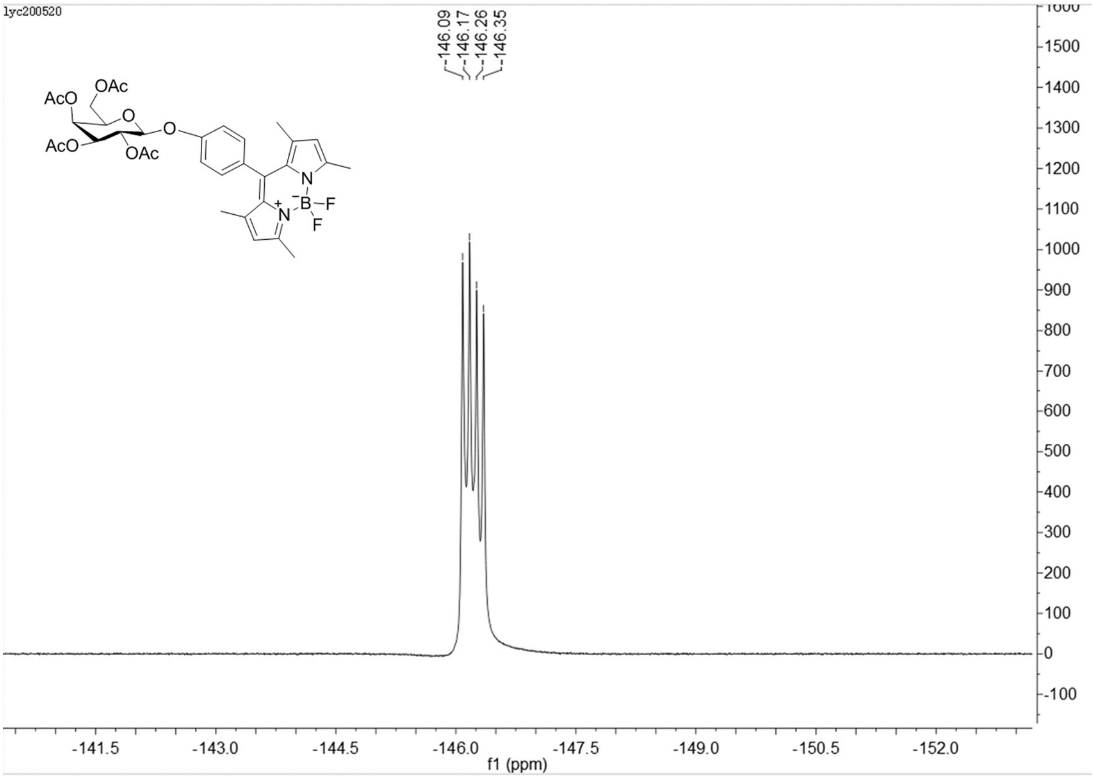

Figure S10 ^{19}F NMR spectrum of compound **3** in $CDCl_3$

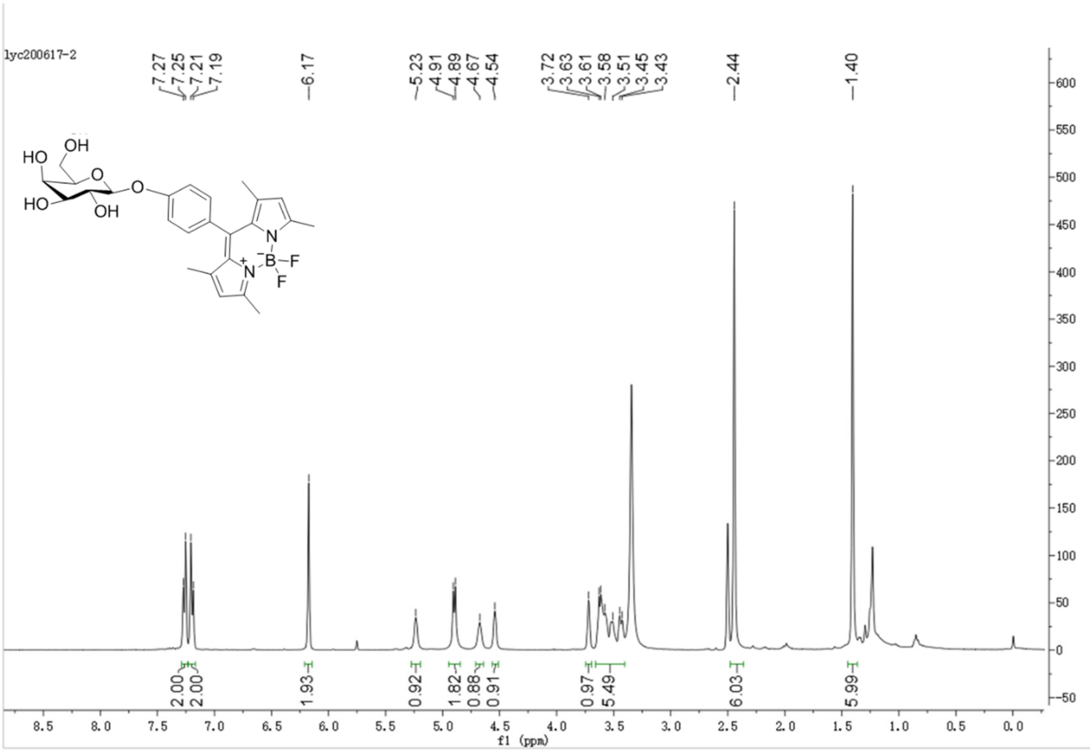

Figure S11 ^{1}H NMR spectrum of BOD-Gal in DMSO-d_6

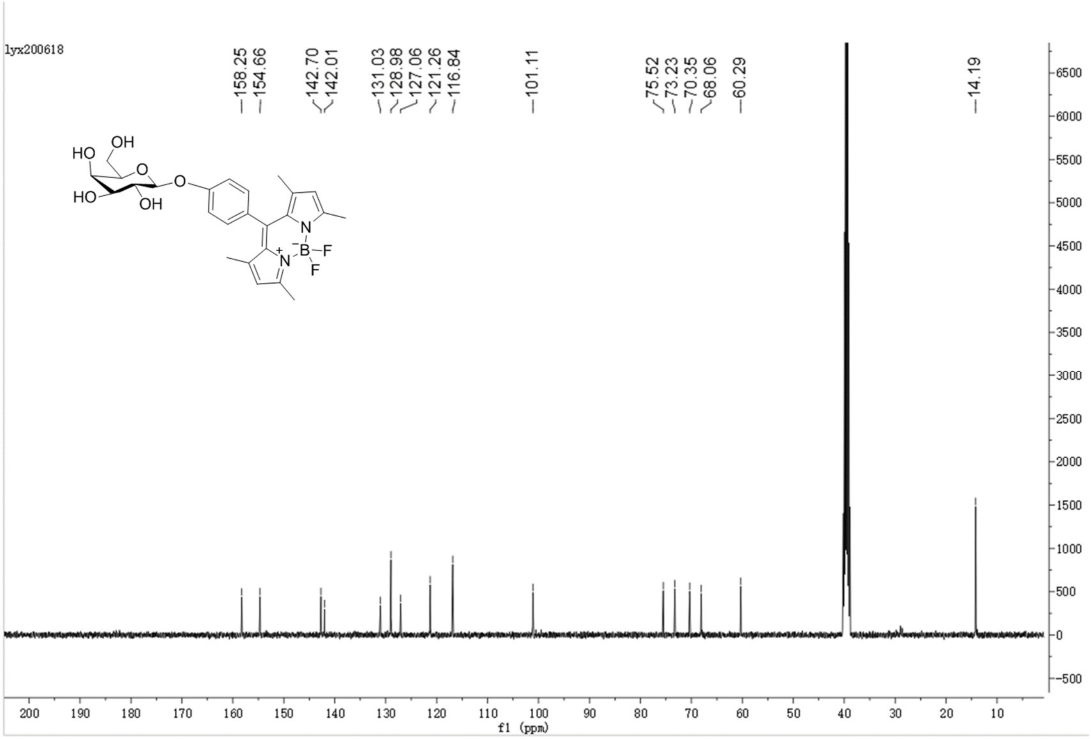

Figure S12 ^{13}C NMR spectrum of BOD-Gal in DMSO-d_6

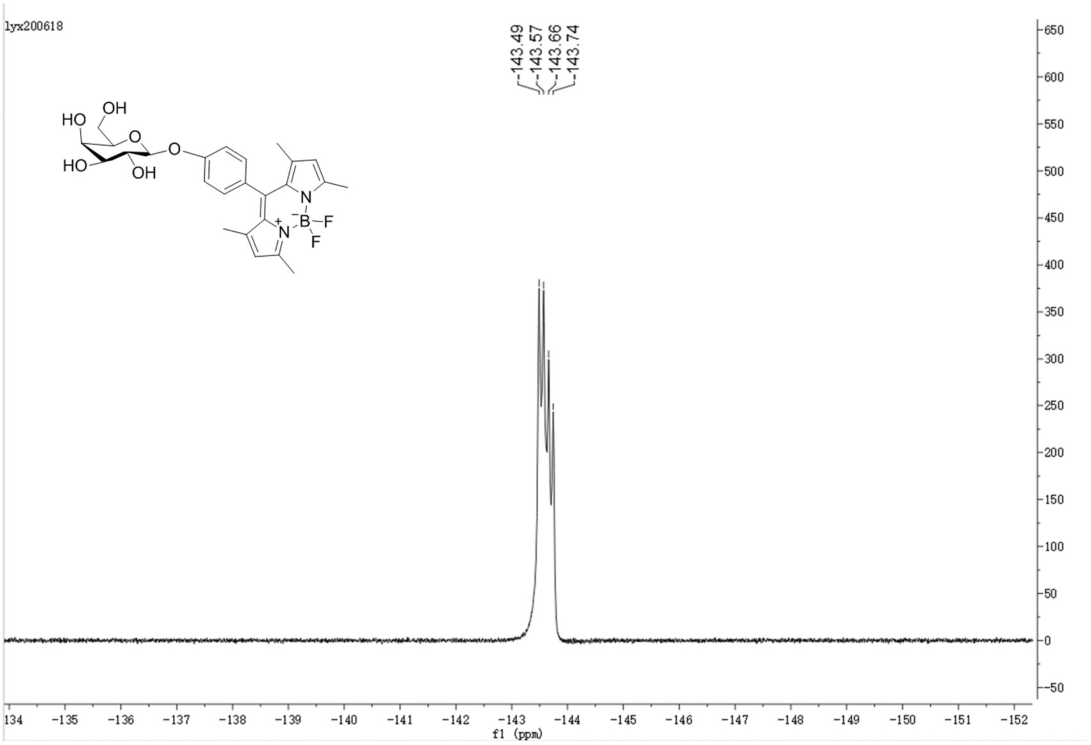

Figure S13 ^{19}F NMR spectrum of BOD-Gal in DMSO-d_6.
